# RNA flow cytometric FISH for investigations into HIV immunology, vaccination and cure strategies

**DOI:** 10.1186/s12981-017-0171-x

**Published:** 2017-09-12

**Authors:** Amy E. Baxter, Julia Niessl, Antigoni Morou, Daniel E. Kaufmann

**Affiliations:** 1 Research Centre of the Centre Hospitalier de l’Université de Montréal (CRCHUM), and Université de Montréal, Montreal, QC Canada; 2 Center for HIV/AIDS Vaccine Immunology and Immunogen Discovery (CHAVI-ID), La Jolla, CA USA

**Keywords:** CD4 T cells, Cytokines, HIV, Vaccine, RNA, Reservoirs, Latency, Single-cell techniques, Flow cytometry, Fluorescence in situ hybridization (FISH)

## Abstract

Despite the tremendous success of anti-retroviral therapy (ART) no current treatment can eradicate latent HIV reservoirs from HIV-infected individuals or generate, effective HIV-specific immunity. Technological limitations have hampered the identification and characterization of both HIV-infected cells and HIV-specific responses in clinical samples directly ex vivo. RNA-flow cytometric fluorescence in situ hybridisation (RNA Flow-FISH) is a powerful technique, which enables detection of mRNAs in conjunction with proteins at a single-cell level. Here, we describe how we are using this technology to address some of the major questions remaining in the HIV field in the era of ART. We discuss how CD4 T cell responses to HIV antigens, both following vaccination and HIV infection, can be characterized by measurement of cytokine mRNAs. We describe how our development of a dual HIV mRNA/protein assay (HIV^RNA/Gag^ assay) enables high sensitivity detection of very rare HIV-infected cells and aids investigations into the translation-competent latent reservoir in the context of HIV cure.

## Background

In spite of remarkable progress over the last 30 years, key challenges remain for the HIV/AIDS field. Persons infected with HIV require life-long anti-retroviral therapy (ART) to suppress viral replication, as the virus survives long-term in the form of latent HIV viral reservoirs [1] (VR). A functional cure is needed to treat these individuals and remove the financial and social burdens of long-term ART. In addition, the major hope to limit the spread of the epidemic, an effective HIV vaccine, remains elusive. We propose that the in-depth study of HIV infection in HIV-positive subjects is required to reach these two goals. Firstly, although the phenomenon of latency is well accepted, there remains a limited understanding of cells that support active HIV replication in ART-naïve subjects and serve as long-lived latent VR from which the virus can rebound in treated persons. Secondly, HIV-infected individuals can raise adaptive and humoral responses to the virus [2–4], which are highly inter-related [5, 6]. Studying how these HIV-specific responses vary between individuals, particularly in cases where HIV replication is naturally controlled, and how they compare to responses elicited by other pathogens or vaccines, provides valuable information for prophylactic HIV vaccine design. Lastly, bringing these two themes together, understanding the interactions between the VR and the individual’s immune system is critical for the development of therapeutic vaccines capable of eliminating HIV-infected (HIV^+^) cells in the cure context.

Here, we discuss how RNA-flow cytometry can be used to advance these key areas of HIV research. This fluorescence in situ hybridization (FISH)-based technique combines branched double-stranded (ds) DNA technology for the detection of viral or host-specific mRNAs with concurrent antibody staining for phenotypic markers (RNA Flow-FISH). Briefly, samples are initially processed as for standard flow cytometry including viability stains, surface and intracellular antibody staining. To detect mRNAs, target sequences are recognized and bound by specific dsDNA target probes along the mRNA. This initial signal is then amplified and the amplified signal is labelled with fluorescent probes. The use of multiple rounds of amplification drastically increases the sensitivity and specificity of RNA Flow FISH, however these aspects are dependent on multiple factors, including the copy number of the target mRNA and the number of target probe pairs used per mRNA. Therefore such features should be determined for each specific experiment/target mRNA (see [7, 8] for details). Samples can be acquired on standard cytometer, with two lasers required as a minimum to enable protein and mRNA detection. Detailed information regarding flow cytometry panels and further advanced technical information can be found in [9]. Importantly, mRNA and protein expression information is acquired at a single-cell level with a high through-put, enabling the identification of very rare events and identification of gene expression patterns in heterogeneous populations.

## How does the immune system respond to HIV?

HIV-infected individuals mount detectable T cell-driven responses to the virus that may be important components of viral control [2]. Yet, in the only trial showing protection to date (RV144), vaccine-induced T cell responses were highly variable and did not correlate with protection [10]. However, the HIV-specific responses detected in these studies are defined and limited by the assays available to monitor them. Population-level techniques, such as Luminex bead arrays, provide only an overview of the response, masking crucial single-cell information. Activation-induced marker (AIM)-style assays [11, 12] detect a heterologous antigen-responsive population at the single-cell level, but alone provide limited functional information. Intracellular cytokine staining (ICS) enables single-cell analysis but requires the addition of protein trafficking inhibitors that may affect concurrent cell phenotyping; and furthermore a number of important CD4 T cell (CD4) cytokines stain poorly in ICS. Thus, cell populations of exceptional interest to the HIV field may be missed by such assays, with antigen-specific T follicular helper cells (Tfh) as a prime example.

The generation of broadly neutralizing antibodies (bnAbs) is assumed critical for a successful HIV prophylactic vaccine strategy [13]. Within the germinal center (GC), Tfh “help” B cells by driving the affinity maturation essential for bnAb development; and, in HIV-infected individuals, a subset of circulating memory Tfh-like cells (cTfh) is associated with such bnAb development [14]. Thus, understanding the generation and function of HIV-specific Tfh and cTfh is key. However, these rare cells are challenging to study; they are often missed by antibody-based assays, as they produce limited amounts of classical Th1/2 cytokines; and only low levels of CXCL13 and IL-21. In contrast, the amplification strategy employed by RNA Flow-FISH enables high sensitivity detection of rare cytokine mRNAs, in our hands identifying IL-21 mRNA^+^ CD4 in the context of idiopathic thrombocytopenic purpura [7]. We are currently studying how the function of HIV-specific cTfh generated in infection varies with viral control, and how this compares to cTfh elicited by other chronic infections (e.g. CMV) or vaccinations (e.g. HBV). Such information will inform development of bnAb-eliciting vaccines attempting to harness this Tfh response.

While we have focused on Tfh cytokines here, it is worth noting that other groups have used this technique to detect alternative antigen-specific responses of interest including IL-17A [15] and IL-10 [16].

## Where does the virus hide?

The major source of HIV during chronic infection has long been recognized as CD4 T cells. The precise characterization of CD4 subsets harbouring replication-competent virus (i.e. provirus contributing to spreading infection) is crucial for the development of therapeutic vaccine or cure strategies targeting these VR; however studies have been limited by the strategies used to detect HIV^+^ cells. For example, in vitro infection studies are limited by the requirement for cellular activation, which alters cell characteristics, preventing phenotyping. Viral DNA measurement by PCR for total or integrated viral genes [17–19] and other assays performed on bulk cell subsets provide the VR size at a population, not single-cell, level; and limiting dilution strategies including the Quantitative Viral Outgrowth Assay (QVOA) [20, 21] and Tat/Rev Limiting Dilution assay (TILDA) [22] provide VR cell frequencies but do not allow phenotypic characterization of the infected cells. Therefore the relative contribution of distinct subsets can only be inferred by pre-sorting cell populations of interest prior to running the assay. This complexity is further confounded by the high prevalence of defective HIV genomes [23]; thus, PCR-based techniques represent a maximal estimate for VR size [24] while QVOA represents a minimal estimate [23]. Therefore, a technique was required that enabled single-cell phenotyping of the HIV^+^ cells that were likely to contain non-defective, functional proviruses.

To address this need, we designed and developed an RNA flow-FISH protocol enabling concurrent detection of HIV Gag protein and GagPol mRNA—the HIV^RNA/Gag^ assay [8]. As cells must contain a provirus capable of producing both HIV mRNAs and protein to be detected (the ‘*translation*-*competent reservoir*’), these cells are more likely to contain replication-competent virus than those cells detected by a DNA-based measure only. Crucially, individual HIV^+^ cells can be concurrently phenotyped for population markers, or additional proteins of interest—a key advantage compared to the techniques described above. The HIV^RNA/Gag^ assay is 1000-fold more specific than standard Gag protein staining and is linear in the range of 1 HIV^RNA+/Gag+^ CD4 per million. These features enable the assay to be used for the detection and phenotyping of HIV^+^ cells, in clinical samples from HIV-infected individuals, directly ex vivo.

Using the HIV^RNA/Gag^ assay, we have begun to determine which CD4 subsets contain the translation-competent reservoir in subjects with chronic viral infection, and thus aim to elucidate in detail where the virus is hiding [8]. We confirmed several observations made using alternative techniques, such as the dominance of memory (both central/transitional and effector) CD4 within the HIV^+^ population [25]. Interestingly, we observed that while HLA-ABC was more likely to be down-regulated on HIV^RNA+/Gag+^ cells as previously reported, HLA-DR was up-regulated in contrast to results obtained with in vitro infection. Furthermore, we determined that cTfh were preferentially infected, supporting seminal studies identifying GC Tfh as an immune-privileged reservoir [26, 27] and found that HIV^RNA+/Gag+^ CD4 were enriched in expression of multiple inhibitory receptors including PD-1, TIGIT and CTLA-4. Thus RNA Flow-FISH can be used to address a key knowledge gap in the detailed identification of VR.

## Monitoring HIV cure strategies

Despite its tremendous success, ART does not represent a cure. HIV is able to persist in the form of a latent reservoir from which the virus can rebound when a patient discontinues therapy. The identification, quantification and monitoring of the size of this reservoir presents a key challenge for HIV cure research. As mentioned above, multiple techniques are currently used, but caveats remain. We have used the HIV^RNA/Gag^ assay to quantify the translation-competent latent reservoir in virally-suppressed, ART-treated individuals at a single-cell level, identifying rare HIV^RNA+/Gag+^ CD4 following maximal stimulation in vitro (median ~4 per million, [8]). Interestingly, we also identified very rare HIV^RNA+/Gag+^ in the absence of stimulation in a subset of samples from these individuals with suppressed plasma viremia (median ~1 per million in samples where events were detected). Whether this finding corresponds to limited viral reactivation or isolated HIV Gag protein/GagPol mRNA production in the absence of complete viral replication remains to be determined. Indeed, the implication that ongoing infection and spread could occur in the presence of suppressive ART is exceptionally controversial [28–30].

“Shock and kill” strategies, whereby latency-reversing agents (LRAs) “shock” the latent VR into reactivating, such that HIV^+^ cells can be targeted by the host’s immune system have been advanced as a possible cure approach. Some LRAs have shown promise in vitro [31] and have already been tested in limited clinical trials [32], however the relative ability of LRAs to induce different cellular VR remains poorly understood. To address this, we have used the HIV^RNA/Gag^ assay to measure latency reversal in vitro by the PKC agonists bryostatin and ingenol. We found that both LRAs induced reactivation with similar efficiencies. Interestingly however, we observed that while ingenol induced reactivation in both the central/transitional and effector memory subsets, bryostatin preferentially reactivated translation-competent virus from the effector memory population [8]. This suggests that LRAs may have differential effects on distinct CD4 subsets; crucial information that would have been missed with population-level assays. Thus, the HIV^RNA/Gag^ assay enables advanced, single-cell monitoring of VR, providing unique knowledge to guide cure strategies.

## Summary

We have described here our use of RNA Flow-FISH to investigate interactions of HIV with peripheral CD4 T cells (both as a HIV reservoir and as an immune cell responding to viral antigen). However, the technique can easily be adapted to investigate tissues, alternative cell types including macrophages, for detection of multiple viral mRNAs and additional host factors. In summary, RNA Flow-FISH represents a powerful, highly versatile technique that has multiple applications for the broader HIV field.
